# State Safety Net Policies and Health of ADRD Caregivers: Evidence from BRFSS and SPPD

**DOI:** 10.1093/geroni/igaf122.3656

**Published:** 2025-12-31

**Authors:** Talha Ali, Siyao Lu, Julie Strominger, Erin Kent, Toni Antonucci, Wassim Tarraf

**Affiliations:** Tufts University, Medford, Massachusetts, United States; Yale University, New Haven, Connecticut, United States; The Ohio State University, Columbus, Ohio, United States; University of North Carolina at Chapel Hill, Chapel Hill, North Carolina, United States; University of Michigan, Ann Arbor, Michigan, United States; Wayne State University, Detroit, Michigan, United States

## Abstract

Caregivers of individuals with Alzheimer’s Disease and Related Dementias (ADRD) experience substantial physical and mental health burdens, often shaped by structural factors such as policy environments. This study examines whether state safety net policies—Medicaid expansion, Medicaid generosity, unemployment benefits, and paid sick leave (PSL)—affect the health of ADRD caregivers, and whether these associations differ by caregiving complexity and sex. We identified ADRD caregivers across 47 states from the U.S. Behavioral Risk Factor Surveillance System (2021–2022; N = 12,025 representing 7,480,376 nationally) and obtained policy data from the State Policy and Politics Database (2020). Outcomes included caregivers’ self-reported poor physical and mental health days in the last month. Negative binomial regression models assessed policy associations with healthy days and tested interactions by caregiving complexity and sex. Northeastern and West Coast states ranked higher in policy generosity, and most Southern and some Midwestern states ranked lower. More generous policies were associated with 0.1-0.5 fewer poor physical and 0.2-0.5 fewer poor mental health days per month among ADRD caregivers, though effects were not statistically significant. Greater Medicaid generosity, unemployment benefits, and PSL were linked to 1–3 and 2–6 fewer days of poor physical and mental health, respectively, among caregivers providing personal care (not significant). However, caregivers providing high care hours without personal care experienced 7–13 more poor mental health days in states with vs. without PSL and Medicaid expansion. No significant sex differences were observed. This suggests targeted state policy efforts are needed to address health risks faced by ADRD caregivers providing high-intensity care.

